# Supplementary material for: Polypropylene Recovery and Recycling from Mussel Nets
Source: Polymers (Basel). 2022 Aug 25;14(17):3469. doi: 10.3390/polym14173469 (PMC9460901; doi:10.3390/polym14173469)
Supplement: Supplementary file 1 [file polymers-14-03469-s001.zip › polymers-1866400-supplementary.pdf]

SUPPLEMENTARY MATERIALS (Loris Pietrelli)

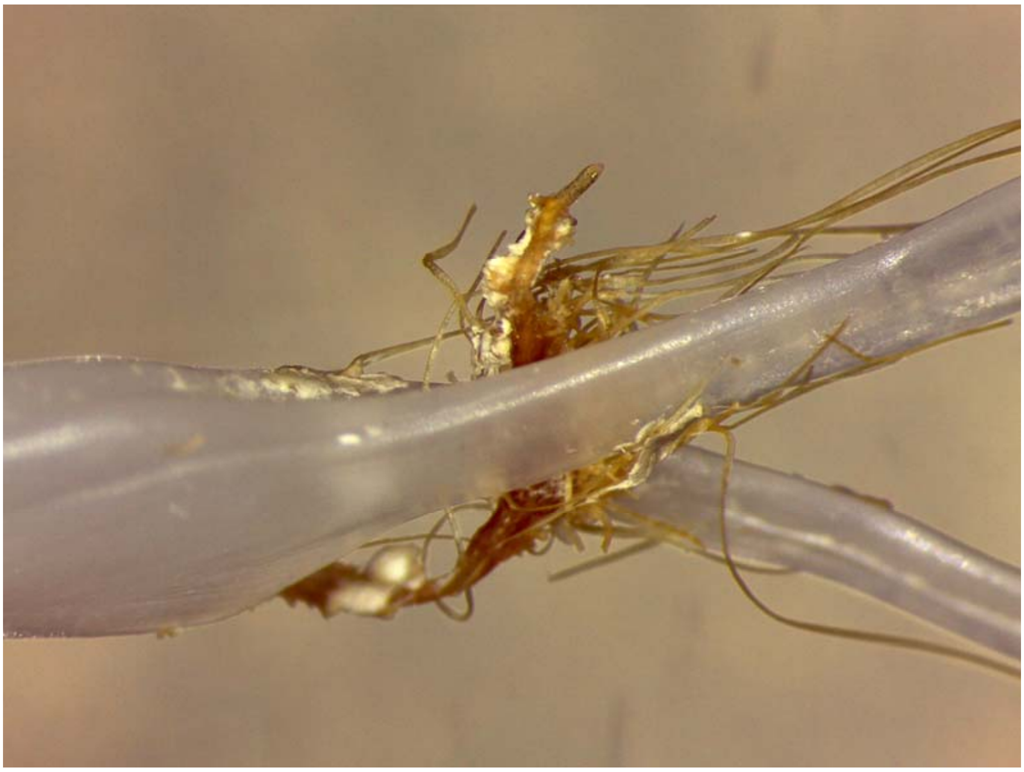

Figure S1. Byssus fibres on the sock surface

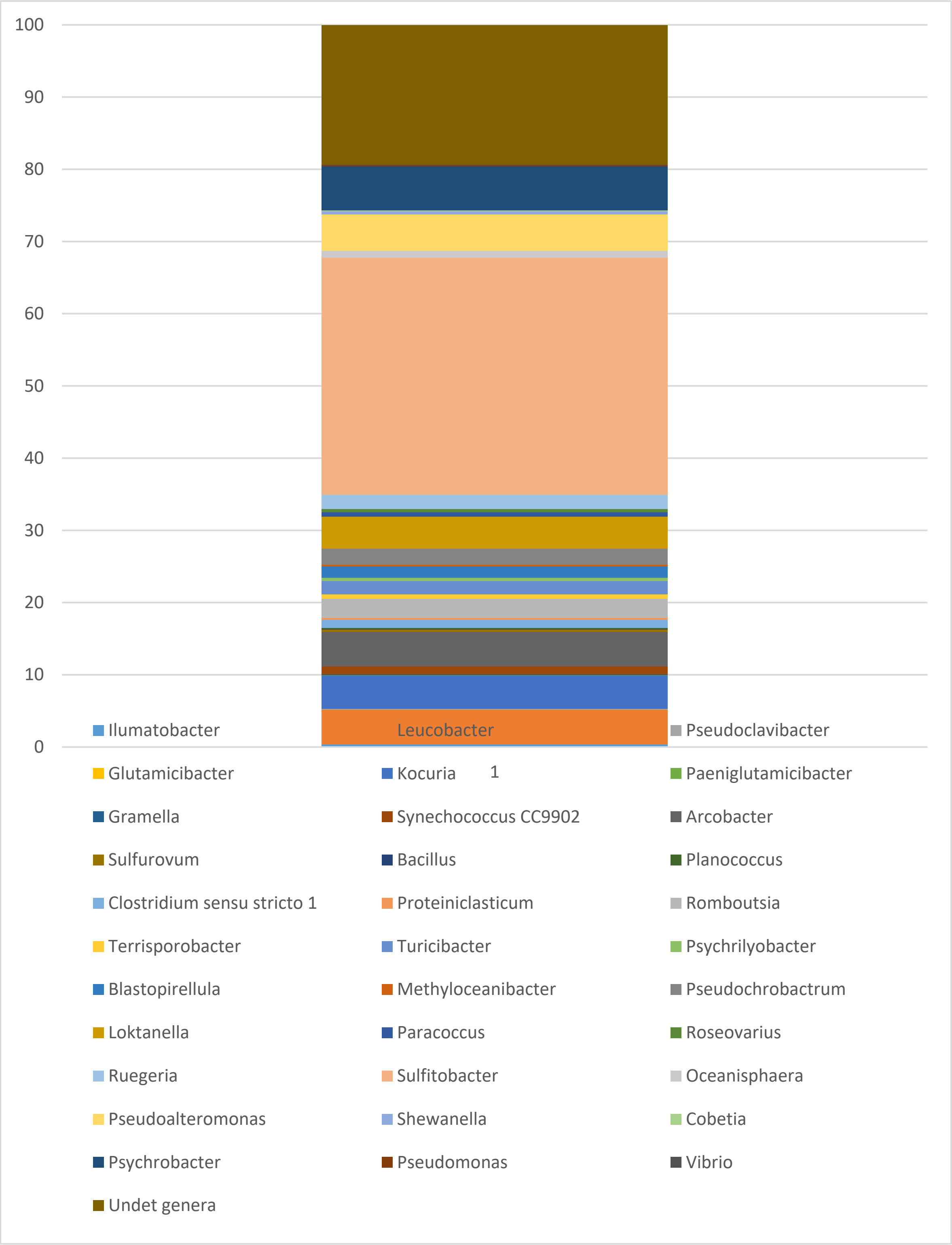

Figure S2. Characterization of the microbial community
